# Technical tip: endoscopic internalization by cutting the drainage tube after endoscopic ultrasound-guided naso-gallbladder drainage for acute cholecystitis

**DOI:** 10.1055/a-2505-9378

**Published:** 2025-01-16

**Authors:** Noriyuki Hirakawa, Shuntaro Mukai, Takayoshi Tsuchiya, Reina Tanaka, Ryosuke Tonozuka, Takao Itoi

**Affiliations:** 1 13112 Department of Gastroenterology and Hepatology, Tokyo Medical University, Tokyo, Japan

Although a fully covered metal stent (FCMS) placed for malignant distal biliary obstruction can maintain long-term patency, it can obstruct the bifurcation of the cystic duct 1 2 . Endoscopic ultrasound-guided naso-gallbladder drainage (EUS-NGBD) for acute cholecystitis caused by FCMS is reported to be effective 3 4 . To prevent recurrence, it is recommended that internalization be performed by cutting the drainage tube in the stomach with a loop cutter after improvement of cholecystitis 5 . Here, we present a case of acute cholecystitis caused by FCMS placement in which EUS-NGBD was performed and endoscopic internalization was achieved using a reusable loop cutter.

A 74-year-old man was referred to us with obstructive jaundice caused by unresectable pancreatic head cancer ( Fig. 1 ). Endoscopic transpapillary drainage was performed by placing a 10-mm FCMS for malignant distal biliary obstruction. Acute cholecystitis occurred when the cystic duct bifurcation became obstructed by the FCMS. Use of an apposition stent to drain the gallbladder represents one option in this situation. However, this approach requires tract dilation, which increases the risk of infected bile leaking into the abdominal cavity. Therefore, EUS-NGBD was performed ( Fig. 2 ). The gallbladder was punctured from the duodenal bulb using a 19-gauge needle under EUS guidance ( Fig. 3 **a** , Video 1 ). A 0.025-inch guidewire was inserted into the gallbladder, and the tract was dilated using a hard-type ultra-tapered bougie dilator. A 5-Fr naso-gallbladder drainage tube was inserted into the gallbladder ( Fig. 3 **b** ). Aspiration of infected bile from the drainage tube led to rapid improvement of inflammation. Endoscopic internalization using a reusable loop cutter was performed 4 days later. The tip of the loop cutter blade was sharpened by biting the edge of a sheet of aluminum foil seven times ( Fig. 4 ). The NGBD tube was cut using the sharpened loop cutter ( Fig. 5 ). No procedure-related adverse events occurred, and chemotherapy was initiated.

**Fig. 1:**
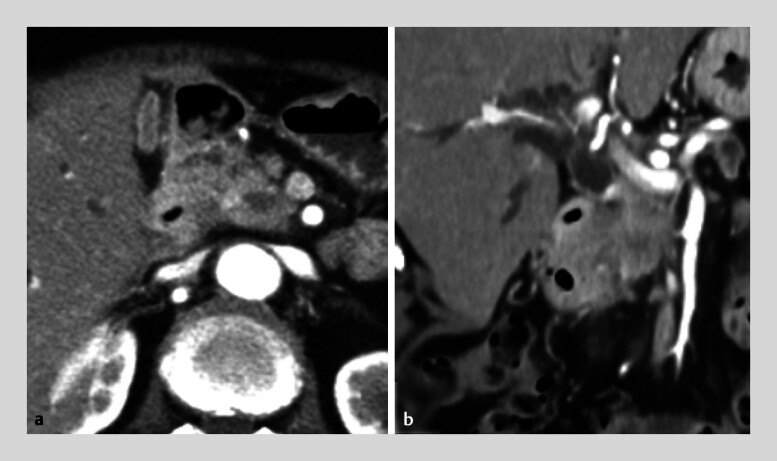
Contrast-enhanced computed tomography scans showing a hypovascular tumor in the pancreatic head. **a** Axial view. **b** Coronal view.

**Fig. 2:**
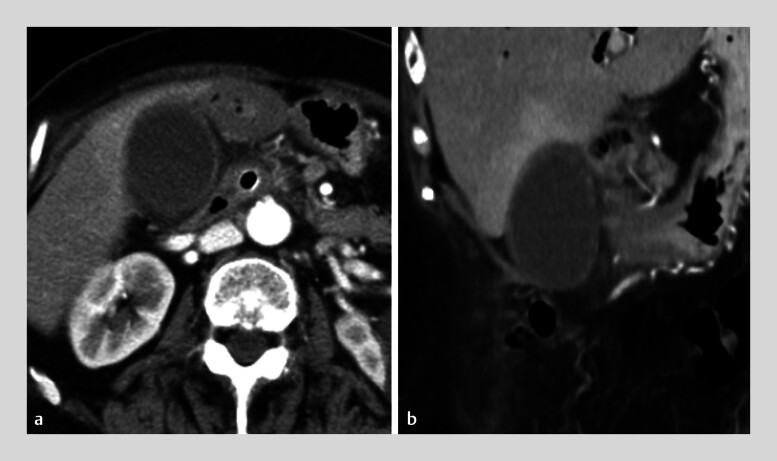
Contrast-enhanced computed tomography scans showing gallbladder enlargement. **a** Axial view. **b** Coronal view.

**Fig. 3:**
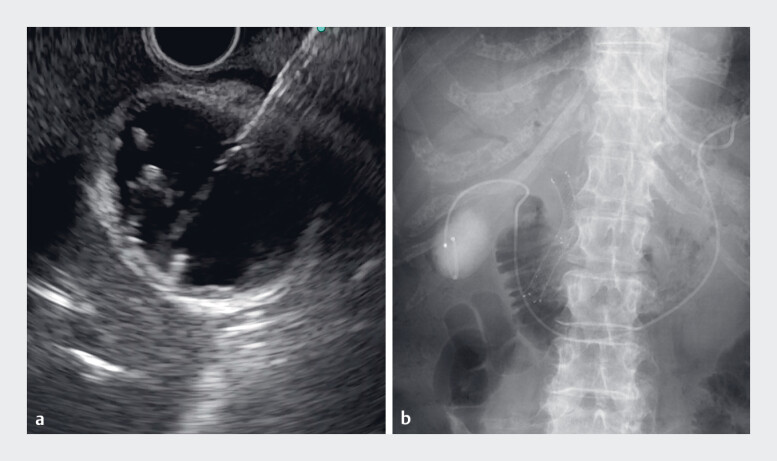
Endoscopic ultrasound (EUS)-guided naso-gallbladder drainage procedure. **a** The gallbladder was punctured from the duodenal bulb using a 19-gauge needle. **b** A transmural naso-gallbladder drainage tube was placed under EUS guidance.

**Fig. 4:**
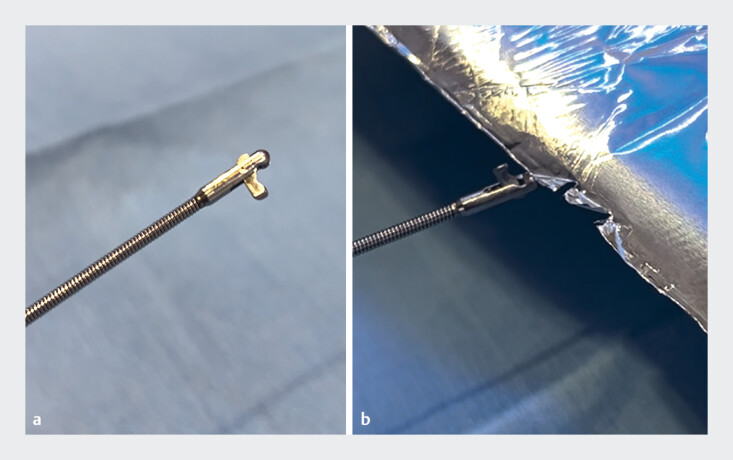
The tip of the blade on the loop cutter was sharpened by biting the edge of a sheet of aluminum foil. **a** The loop cutter. **b** The sharpening procedure.

**Fig. 5:**
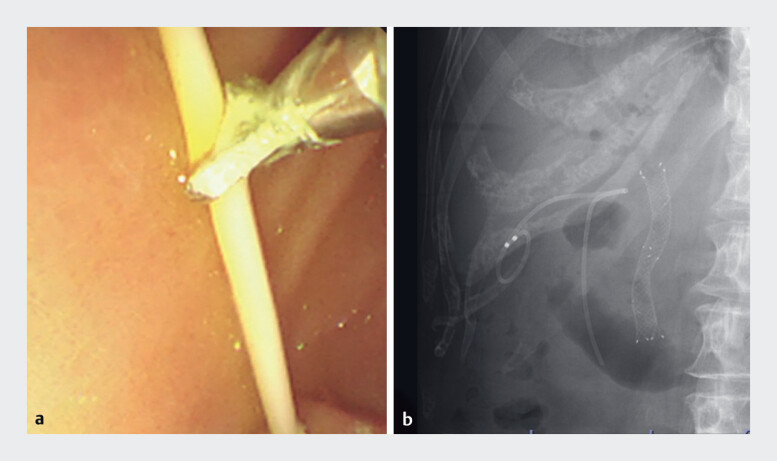
Endoscopic internalization by cutting a transmural naso-gallbladder drainage tube under endoscopic ultrasound guidance. **a** Endoscopic view. **b** Fluoroscopic image.

Endoscopic internalization by cutting a drainage tube after endoscopic ultrasound-guided naso-gallbladder drainage for acute cholecystitis.Video 1

Endoscopy_UCTN_Code_TTT_1AS_2AH

## Notes

Endoscopy E-Videos https://eref.thieme.de/e-videos
*E-Videos* is an open access online section of the journal *Endoscopy* , reporting on interesting cases and new techniques in gastroenterological endoscopy. All papers include a high-quality video and are published with a Creative Commons CC-BY license. Endoscopy E-Videos qualify for HINARI discounts and waivers and eligibility is automatically checked during the submission process. We grant 100% waivers to articles whose corresponding authors are based in Group A countries and 50% waivers to those who are based in Group B countries as classified by Research4Life (see: https://www.research4life.org/access/eligibility/ ). This section has its own submission website at https://mc.manuscriptcentral.com/e-videos .
